# The W195 Residue of the Newcastle Disease Virus V Protein Is Critical for Multiple Aspects of Viral Self-Regulation through Interactions between V and Nucleoproteins

**DOI:** 10.3390/v16040584

**Published:** 2024-04-10

**Authors:** Qiaolin Wei, Wenbin Wang, Fanxing Meng, Ying Wang, Ning Wei, Jianxia Tian, Hanlue Li, Qiqi Hao, Zijie Zhou, Haijin Liu, Zengqi Yang, Sa Xiao

**Affiliations:** 1 College of Veterinary Medicine, Northwest A&F University, Yangling, Xianyang 712100, China; icemey@163.com (Q.W.); fanxing_meng@163.com (F.M.); 15829243198@163.com (Y.W.); w244728911@163.com (N.W.); 18209507259@163.com (J.T.); 2015011055@nwafu.edu.cn (H.L.); haoqiqi@nwafu.edu.cn (Q.H.); 13571865032@163.com (Z.Z.); liuhaijin@nwafu.edu.cn (H.L.); yzq1106@nwsuaf.edu.cn (Z.Y.); 2 Poultry Institute, Shandong Academy of Agricultural Science, Jinan 250100, China; wangwenbin1230@163.com

**Keywords:** Newcastle disease virus, V protein, minigenome, inclusion body formation

## Abstract

The transcription and replication of the Newcastle disease virus (NDV) strictly rely on the viral ribonucleoprotein (RNP) complex, which is composed of viral NP, P, L and RNA. However, it is not known whether other viral non-RNP proteins participate in this process for viral self-regulation. In this study, we used a minigenome (MG) system to identify the regulatory role of the viral non-RNP proteins V, M, W, F and HN. Among them, V significantly reduced MG-encoded reporter activity compared with the other proteins and inhibited the synthesis of viral mRNA and cRNA. Further, V interacted with NP. A mutation in residue W195 of V diminished V–NP interaction and inhibited inclusion body (IB) formation in NP-P-L-cotransfected cells. Furthermore, a reverse-genetics system for the highly virulent strain F48E9 was established. The mutant rF48E9-V_W195R_ increased viral replication and apparently enhanced IB formation. In vivo experiments demonstrated that rF48E9-V_W195R_ decreased virulence and retarded time of death. Overall, the results indicate that the V–NP interaction of the W195 mutant V decreased, which regulated viral RNA synthesis, IB formation, viral replication and pathogenicity. This study provides insight into the self-regulation of non-RNP proteins in paramyxoviruses.

## 1. Introduction

Newcastle disease virus (NDV) causes highly contagious respiratory, intestinal and neurological diseases in avian species. NDV, also known as avian paramyxovirus serotype 1 or avian avulavirus 1, is a linear, nonsegmented, negative-strand RNA virus that belongs to the genus *Orthoavulavirus* of the subfamily *Avulavirinae* in the family *Paramyxoviridae* [1,2]. The genome of NDV encodes six structural proteins: nucleoprotein (NP), phosphoprotein (P), matrix protein (M), fusion protein (F), hemagglutinin–neuraminidase (HN) and large polymerase protein (L) [3]. The order of the NDV genome is 3′Leader-NP-P-M-F-HN-L-trailer5′. Each gene contains conserved transcriptional regulatory sequences known as gene starts (GSs) at the 3′ end and gene ends (GEs) at the 5′ end, which are used as transcriptional start and polyadenylation stop signals, leading to a gradient of mRNA populations [4,5]. There are short, nontranscribed, intergenic sequences (IGSs) between the open reading frame of each gene and the GS and GE regions [6,7]. In general, the NDV genome has three sizes—15,186, 15,192 or 15,198 nucleotides (nt)—all of which follow the “rule of six” [4,8].

Viral transcription and replication rely on the viral ribonucleoprotein (RNP) complex, which is composed of the viral proteins NP, P and L as well as viral RNA in NDV. The NP is the most abundant protein in viral particles. It encapsulates genomic RNA via NP–NP and NP–RNA interactions and forms a left-handed coil helical core [4,5,9]. The P protein, as a bridge, mediates the interaction between the L protein and the NP–RNA template and stabilizes the L protein in the P–L complex, which acts as a viral RNA-dependent RNA polymerase (RdRp) to form an active RNP [10]. The viral RNPs are located in specialized intracellular compartments in infected host cells, called inclusion bodies (IBs), viral factories or viroplasm, where transcription and replication of the viral genome take place in many negative-strand RNA viruses [11]. IB formation can be observed in paramyxoviruses, such as NDV [12], mumps virus (MuV) [13], Nipah virus (NiV) [14] and human parainfluenza virus type 3 (HPIV3) [15]. The NP and P of NDV are the minimal components necessary for IB formation [12].

NDV produces two accessory proteins, V and W, via RNA editing of the P gene [16]. V is multifunctional in viruses and hosts. It is crucial for its ability to antagonize the innate immune system of host cells [17,18]. It also plays a vital role in host range restriction of NDV to efficiently prevent innate host defenses, such as the IFN response and apoptosis [19]. Moreover, V, as a viral regulator of viral replication and transcription, inhibits viral RNA synthesis in several paramyxoviruses, including HPIV5, measles virus (MeV), NiV, Sendai virus (SeV) and simian virus 5 (SV5) [20,21,22,23,24]. The inhibition of HPIV5 V and SeV V is associated with the interaction between the NP and V proteins, while the inhibitory effect of the HPIV2 V protein on GFP expression in a minigenome system is mediated by binding to the L protein, not by NP binding [20,23,25]. However, the role of the V of NDV as a viral self-regulator in transcription and replication is unknown. In other virus families, matrix proteins of Ebola virus VP24 and VP40 can regulate viral replication and transcription [26,27].

In this study, we established two NDV systems, minigenome (MG) and reverse genetics. Using these platforms, viral non-RNP proteins were screened for viral transcription and replication. V was identified as a viral self-regulator in this process. Residue W195 of V was identified as crucial for the interaction of V and NP, which regulated viral RNA synthesis, IB formation, viral replication and pathogenicity both in vitro and in vivo. This study provides a precise understanding of the self-regulation of V in paramyxoviruses.

## 2. Materials and Methods

### 2.1. Ethics Statement

Nine- to 11-day-old SPF embryonated chicken eggs and 3-week-old SPF chickens were purchased from Jinan Spafas Poultry Co., Ltd. (Jinan, China). All animal experiments were approved by the Animal Care and Use Committee of Northwest A&F University, China, and were conducted in accordance with guidelines established by the Chinese Committee for Animal Experiments (approval numbers: 2021022).

### 2.2. Cells, Virus and Antibody

DF-1 (ATCC CRL-12203), BHK-21 (ATCC CCL-10) and HEK293T (ATCC CRL-11268) cells were purchased from ATCC and maintained in Dulbecco’s modified Eagle’s medium (DMEM) supplemented with 10% fetal bovine serum (FBS) and 1% penicillin-streptomycin at 37 °C with 5% CO_2_. The NDV F48E9 strain (MG456905.1) and LaSota strain (AF077761.1) were obtained from the China Institute of Veterinary Drug Control (Beijing, China). The F48E9 strain is highly virulent and is commonly used in NDV vaccine challenge experiments in China. The viruses were propagated in 9- to 11-day-old SPF embryonated chicken eggs. Monoclonal antibody (mAb) against HN was prepared in our previous report [28], and preparation of V mAb used the same method. Rabbit anti-NP polyclonal antibody (pAb) was prepared by our laboratory.

### 2.3. Construction of the Minigenome (MG) System and Expression Plasmids

The LaSota strain MG system was constructed. The T7 promoter-Trailer-MCS-Leader-HDV/Rbz-Terminator was synthesized and cloned and inserted into the PUC19 vector. The eGFP gene and firefly luciferase gene were cloned and inserted into multiple cloning sites (MCSs) via SphI and ClaI, respectively, to generate the pUC19-MG-eGFP and pUC19-MG-Fluc plasmids. The plasmids pCAGGS-NP, pCAGGS-P, pCAGGS-L and pCAGGS-T7 were constructed. The pCDNA-2 × Flag-V, pCDNA-2 × Flag-M, pCDNA-2 × Flag-W, pCDNA-2 × Flag-F, pCDNA-2 × Flag-HN and pCDNA-KHA-NP plasmids were constructed using cDNA from LaSota as a template. Truncated V was cloned and inserted into the pCDNA-2 × Flag plasmid. The primers are listed in Table S1 of Supplementary Materials.

### 2.4. MG Assay

BHK-21 cells were seeded in 24-well plates. The following plasmids were cotransfected into the cells: 300 ng of pUC19-MG-eGFP or pUC19-MG-Fluc, 100 ng of pCAGGS-NP, 100 ng of pCAGGS-P, 100 ng of pCAGGS-L and 300 ng of pCAGGS-T7. For the dose-dependent MG assays of viral proteins, increasing amounts of pCDNA-2 × Flag-V, pCDNA-2 × Flag-M, pCDNA-2 × Flag-W, pCDNA-2 × Flag-F and pCDNA-2 × Flag-HN (50 ng, 200 ng, 400 ng, 800 ng) were cotransfected into BHK-21 cells. For the MG assays of mutant V, 50 ng of pCDNA-2 × Flag-V or pCDNA-2 × Flag-V_W195A_ was cotransfected into BHK-21 cells. At 36 h post-transfection, the medium was removed, and the cells were lysed with lysis buffer. Clarified supernatant was obtained after centrifugation at 12,000 rpm for 10 min. Furthermore, an aliquot of 30 µL of supernatant was used to measure luciferase activity with a Spark multimode microplate reader (Tecan, Männedorf, Switzerland), and the relative light ratio was calculated. The relative luciferase activity was normalized to that of Renilla luciferase (Rluc) by transfecting pRL-SV40-N (Beyotime Biotechnology, Shanghai, China).

### 2.5. RT-qPCR

For the analysis of viral genome RNA (vRNA), mRNA and complement RNA (cRNA) amounts in the MG system, the pUC19T-Fluc plasmid was constructed and diluted to 1 × 10^9^~1 × 10^1^ copies·μL^−1^. qPCR was performed using the diluted plasmid as a template to establish a standard curve. The Ct value was substituted into the formula to obtain the standard curve for the Fluc gene. BHK-21 cells were cotransfected with 2 μg of pUC19-MG-Fluc, 1 μg of pCAGGS-NP, 1 μg of pCAGGS-P, 1 μg of pCAGGS-L, 2 μg of pCAGGS-T7 and 1 μg of pCDNA-2 × Flag or pCDNA-2 × Flag-V. At 36 h post-transfection, total RNA was extracted, and 2 μg of RNA was reverse transcribed. The vRNA, cRNA and mRNA levels were detected using specific primers (Table S1 of Supplementary Materials) by qPCR and calculated by a formula. For the analysis of IFN-β levels in NDV-infected cells, DF-1 cells were infected with rF48E9 or rF48E9-V_W195R_ at an MOI of 0.01. Total RNA was extracted at 0, 4, 8 and 12 hpi, and RT-qPCR was performed to detect the level of IFN-β.

### 2.6. Coimmunoprecipitation (CO-IP)

For the analysis of the interaction between the V and NP of NDV, HEK293T cells in 60 mm diameter dishes were transfected with pCDNA-KHA-NP and pCDNA-2 × Flag-V or truncated V at a dose of 2 µg. At 36 h post-transfection, the cells were lysed with 500 µL of ice-cold RIPA lysis buffer supplemented with PMSF (1:1000) for 40 min, and the clarified supernatant was obtained after centrifugation at 12,000 rpm for 10 min at 4 °C. A total of 40 μL of supernatant was obtained for use as input samples. Mouse anti-Flag or anti-HA mAbs (Cell Signaling Technology, Danvers, MA, USA) were added to the remaining supernatant, and the complexes were incubated at 4 °C overnight. Thirty microliters of ice-cold PBS-washed protein A/G beads (Smart-Life Sciences, Changzhou, China) were added to the complexes and incubated at 4 °C overnight. The beads were further washed 3 times with ice-cold PBS, and 40 μL of 1× SDS loading buffer was added to suspend the pellets. The samples were heated at 95 °C for 10 min and subjected to sodium dodecyl sulfate-polyacrylamide gel electrophoresis (SDS-PAGE) for Western blot analysis.

### 2.7. Western Blot

The protein samples were subjected to 12% SDS-PAGE and transferred to a polyvinylidene fluoride (PVDF) membrane. The membranes were blocked with 5% (*w*/*v*) skim milk in Tris-buffered saline (TBS) for 2 h at room temperature and then incubated at 4 °C overnight with the following primary antibodies: mouse anti-Flag mAb, mouse anti-actin mAb, rabbit anti-Flag pAb (Proteintech, Wuhan, China), rabbit anti-HA pAb, mouse anti-HN mAb or rabbit anti-NP pAb. After washing three times with TBST, the membrane was incubated for 1 h at room temperature with HRP-conjugated affinipure goat anti-mouse IgG (H + L) or goat anti-rabbit IgG (H + L) (Proteintech, Wuhan, China) diluted 1:3000. ECL Western blot detection reagents were used to visualize the target signals.

### 2.8. Immunofluorescence Assay (IFA)

For the analysis of the colocalization of V and NP in cells, DF-1 cells were infected with LaSota at an MOI of 0.5. For the analysis of the role of V or V_W195A_ on IBs, DF-1 cells were cotransfected with pCMV-NP-P-L (500 ng) and pCDNA-2 × Flag-V (500 ng) or pCDNA-2 × Flag-V_W195A_ (500 ng). For the analysis of the IB formation in NDV-infected cells, DF-1 cells were infected with rF48E9 or rF48E9-V_W195R_ at an MOI of 0.01. At 36 h post-transfection or 24 h post-infection, the cells were fixed with 4% paraformaldehyde in PBS for 15 min and permeabilized with 0.1% Triton X-100 for 10 min at room temperature. After blocking with PBS containing 1% bovine serum albumin (BSA) for 1 h at 37 °C, the cells were incubated with rabbit anti-NP pAb (1:300), mouse anti-V mAb (1:300) or mouse anti-Flag mAb (1:300) at 4 °C overnight. After being washed with PBS three times, the cells were then incubated with goat anti-mouse IgG H&L (Alexa Fluor® 594) (Abcam, Cambridge, UK), fluorescein (FITC)-conjugated affinipure goat anti-rabbit IgG (H + L), coraLite594–conjugated goat anti-rabbit IgG (H + L) antibody, or fluorescein (FITC)–conjugated affinipure goat anti-mouse IgG (H + L) (Proteintech, Wuhan, China) for 30 min at 37 °C. After being washed 3 times with PBS, the cells were incubated with DAPI (4′,6′-diamidino-2-phenylindole) for nuclear staining. The cells were observed and photographed under a confocal microscope.

### 2.9. Construction of the F48E9 Strain Full-Length Clone

The full-length cDNA of the NDV-F48E9 (GenBank: MG456905.1) genome was constructed in plasmid pBR322, a modified form of plasmid pBR322 in which the fragment between the Asc I and Rsr II sites was removed and replaced by a 122 nt oligonucleotide linker. The 84 nt hepatitis delta virus (HDV) antigenomic nuclease sequence and the T7 RNA polymerase transcription termination signal were inserted into the downstream end of this linker. RNA extracted from NDV-F48E9-infected chicken embryo allantoic fluid was used to generate the full-length cDNA of the complete 15,192 nt genome by RT-PCR. The full length of the genome was constructed as eight fragments. To facilitate construction, five restriction endonuclease sites, Pme I, Pac I, AsiS I, Not I and SnaB I, were created in the 3′ untranslated region (3′-UTR) of the NP, P, M, F and HN genes, respectively. Two enzyme sites, Spe I and Sac II, were created in the L gene. The Rsr II digestion sites on the HN and L gene sequences were deleted by overlapping PCR, and these fragments were subsequently cloned between the T7 promoter of the pBR322 plasmid and the HDV antigenome nuclease sequence. The support plasmids pCAGGS-NP, pCAGGS-P and pCAGGS-L were constructed to express the NP, P and L proteins, respectively. The gene expressing fluorescence (eGFP) was cloned between genes P and M. Gene end, gene start and Kozak sequences were added before the CDS region of the fluorescent gene using primers, and a base was inserted between the gene end and gene start to ensure that the inserted fragment corresponded to the rule of six. The primers are listed in Table S1 of Supplementary Materials.

### 2.10. Rescue of rF48E9, rF48E9-eGFP and rF48E9-V_W195R_

The full-length infectious recombinant plasmid rF48E9-V_W195R_ was constructed. The P gene with a T-to-C mutation at position 582 was amplified through overlapping PCR and cloned and inserted into rF48E9 to replace the rF48E9 P gene using the restriction enzymes PmeI and PacI. BHK-21 cells were grown overnight to 90 to 95% confluence in six-well culture plates and were cotransfected with 2 µg of the respective full-length cDNA plasmid, 0.4 µg of pCAGGS-T7, 0.8 µg of pCAGGS-NP, 0.8 µg of pCAGGS-P and 0.4 µg of pCAGGS-L by using 10 µL of Turbofect (Thermo Scientific, Waltham, MA, USA). The transfection mixture was replaced after 20 h with DMEM containing 2% FBS. Three days after transfection, the BHK-21 cells were scraped into the medium and frozen and thawed three times, and the resulting supernatant was inoculated into the allantoic cavities of 9-day-old embryonated SPF chicken eggs. The allantoic fluid was harvested 3 days post-inoculation and tested for HA activity.

### 2.11. Viral Growth Kinetics

The growth kinetics of the NDV strains F48E9, rF48E9, rF48E9-eGFP and rF48E9-V_W195R_ were determined under multiple growth cycle conditions. DF-1 cells in 6-well culture plates were infected with one of these viruses at an MOI of 0.01. The culture supernatants were collected at 12 h intervals. The viral titers in the collected supernatants were determined through the endpoint method using limiting dilution. The TCID_50_ titer was calculated according to the Reed–Muench method. All experiments were performed in triplicate.

### 2.12. Animal Experiments

The mean death time (MDT) and intracerebral pathogenicity index (ICPI) were measured to assess the virulence of rF48E9 and rF48E9-V_W195R_. Two viruses were diluted and inoculated into 10 SPF chicken embryos at each dilution. The death time of each embryo was recorded. The MDT was determined as the mean time required for the minimum lethal dose of the virus to yield 100% mortality, which was defined as the highest dilution needed to kill all the inoculated embryos. The strains were categorized based on the mean death time (MDT) as follows: MDT of <60 h, virulent strains; MDT of 60 to 90 h, moderately virulent strains; and MDT of >90 h, avirulent strains. The ICPI was determined by calculating the mean score per bird per observation point throughout the 8-day duration. One-day-old SPF chicks were inoculated with 0.05 mL of a 1:10 dilution of fresh allantoic fluid containing rF48E9 or rF48E9-V_W195R_ via the intracerebral route and monitored for clinical symptoms and mortality every 24 h for 8 days. Chicks inoculated with PBS were used as the negative control. Scoring was performed at each observation point as follows: normal (score of 0), sick (score of 1), and dead (score of 2). The pathogenicity of rF48E9 and rF48E9-V_W195R_ was determined in chickens. Nine three-week-old SPF chickens were challenged with 10^5^ pfu of either rF48E9 or rF48E9-V_W195R_ via the intraocular–nasal route and observed for clinical symptoms and mortality daily for 10 days. Chickens inoculated with PBS were used as the negative control. Three chickens from each group were selected for dissection, and various organs were collected for the TCID_50_ assay. The tissues were fixed in phosphate-buffered formalin (4%) and sent to Shaanxi Yike Biotechnology Service Co., Ltd. (Xi’an, China) for the HE assay. The paraffin-embedded tissues were sectioned, deparaffinized, rehydrated and subsequently stained.

### 2.13. Statistical Analysis

All experimental data were analyzed by an unpaired Student’s *t*-test using GraphPad Prism 5. All the data are presented as the means ± standard deviations (SDs) from three independent experiments. *p* < 0.05 was considered to indicate statistical significance.

## 3. Results

### 3.1. V Inhibits Expression of MG-Encoded Reporters

**Figure 1:**
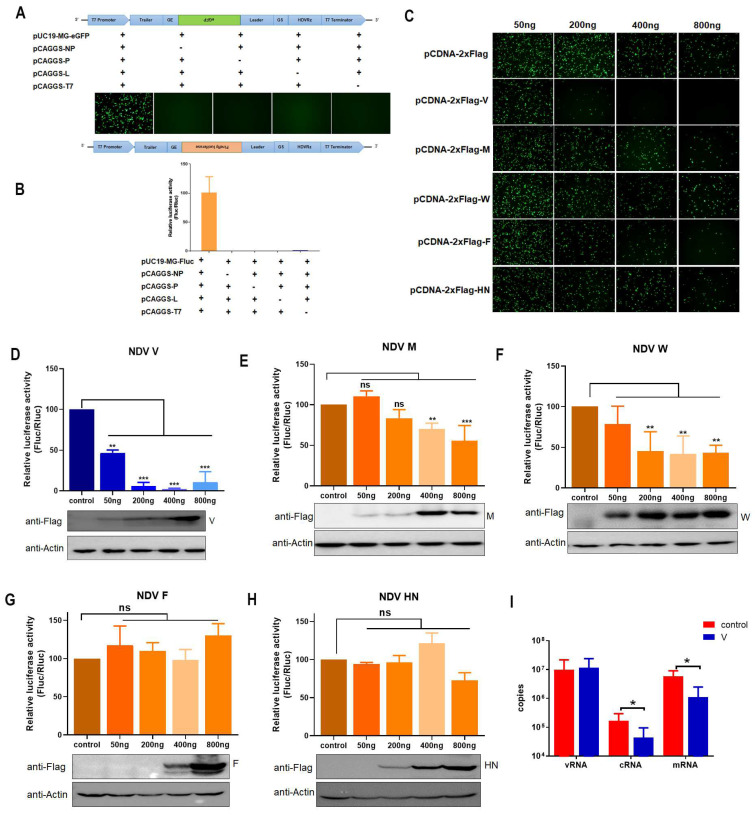
V inhibits MG-encoded reporter expression. (**A**) Schematic representation of the NDV MG system and the expression of the eGFP reporter. Plasmids of MG, T7 were cotransfected into BHK-21 cells for 36 h. (**B**) Schematic representation of the NDV MG system and the activity of the Fluc reporter; relative luciferase activity was analyzed at 36 h post-transfection; scale bar, 100 μM. (**C**) Inhibition of GFP expression by viral non-RNP proteins in MG-eGFP-transfected BHK-21 cells for 36 h. (**D**–**H**) Inhibition of Fluc activity by V, M, W, F and HN at 36 h post-transfection. A dual-luciferase assay was performed to measure luciferase activity. Viral protein expression was determined by Western blot. (**I**) Inhibition of viral RNA synthesis by V. BHK-21 cells were transfected with the MG-Fluc system and V genes. pCDNA-2x Flag was used as a negative control. RNA was extracted, and qPCR was performed at 36 h post-transfection. The results of three independent experiments are shown. Error bars represent standard deviations of the means. NS, not significant; *, *p* < 0.05; **, *p* < 0.01; ***, *p* < 0.001.

To investigate the regulatory role of NDV proteins in transcription and replication in addition to viral RNP-related proteins, two viral MG systems with eGFP and Fluc reporters were established. eGFP and Fluc were separately expressed in BHK-21 cells cotransfected with the NP, P, L and T7 plasmids (Figure 1A,B). In the presence of the viral proteins V, M, W, F and HN, the MG assays showed that the expression of the eGFP reporter was strongly inhibited by the V protein but not by the W, M, F or HN proteins (Figure 1C). Similarly, Fluc activity was dramatically decreased by the V protein in a dose-dependent manner (Figure 1D), while the inhibitory effects of M and W were weaker than that of V (Figure 1E,F), and the F and HN proteins had no effect (Figure 1G,H). For standardizing transfection efficiency, a control vector encoding Rluc under a polymerase II-dependent promoter was cotransfected in all transfection experiments. In addition, to detect viral RNA synthesis, a standard curve for the reporter gene was established by qPCR with formula y = −3.6724x + 39.932, R^2^ = 0.9946. V significantly inhibited mRNA and cRNA synthesis (Figure 1I). These results indicated the inhibitory effects of the V protein on viral transcription and replication.

### 3.2. The Interaction of V with NP Reduces MG-Encoded Reporter Activity

**Figure 2:**
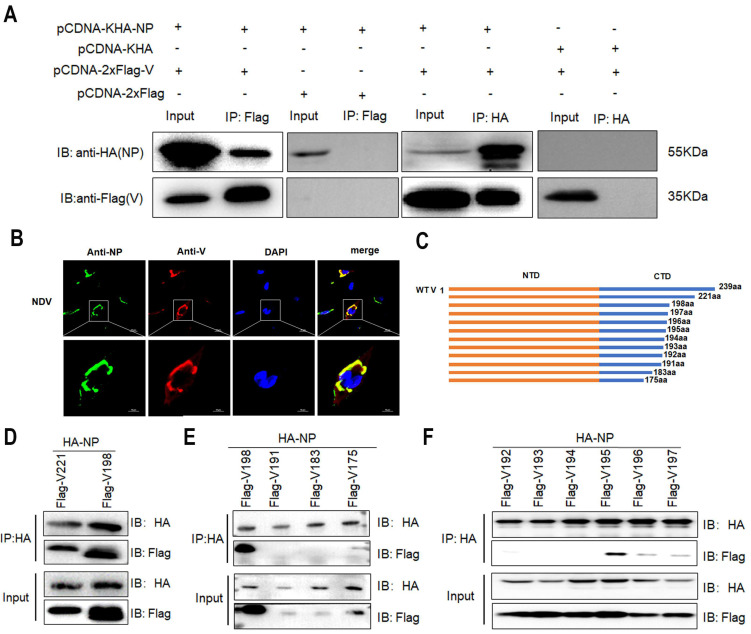
Interactions between NP and V mutants. (**A**) A Co-IP assay was performed on HEK293T cells transfected with plasmids carrying HA-NP and Flag-V. Empty plasmids were used as negative controls. (**B**) Subcellular localization of V and NP in NDV-infected cells. DF1 cells were infected with LaSota (0.5 MOI) and analyzed by IFA at 24 hpi. NP (green), V (red) and nuclei (blue) were observed by confocal microscopy; scale bar, 20 μM. (**C**) Schematic representation of the V truncations. (**D**–**F**) Co-IP analysis of the interaction between NP truncations and V in cotransfected HEK293T cells.

Next, we explored whether the interaction of the V protein with viral RNP-related proteins inhibited viral MG-encoded reporters via coimmunoprecipitation (Co-IP) experiments. The NP and V proteins were coimmunoprecipitated in HEK293T cells cotransfected with plasmids carrying HA-NP and Flag-V (Figure 2A). NP and V were found to be colocalized in NDV-infected cells by an immunofluorescence assay (IFA) (Figure 2B). These results suggested an interaction between V and NP. Furthermore, to identify the specific domain(s) of the V protein involved in the interaction with NP, a series of V deletion mutants were generated and subjected to Co-IP experiments (Figure 2C). The results showed that NP was immunoprecipitated by the N-terminal 1-221, 1-198 amino acids (aa) of the V protein, while 1-191, 1-183 and 1-175 aa did not interact with NP, indicating that the region 191–198 aa of V was crucial for V–NP interaction (Figure 2D,E). Further analysis using V deletion mutants revealed that NP was immunoprecipitated by V1-195 aa, while V1-194 aa did not interact with NP, highlighting the critical role of tryptophan at position 195 (W195) in V–NP interaction (Figure 2F).

**Figure 3:**
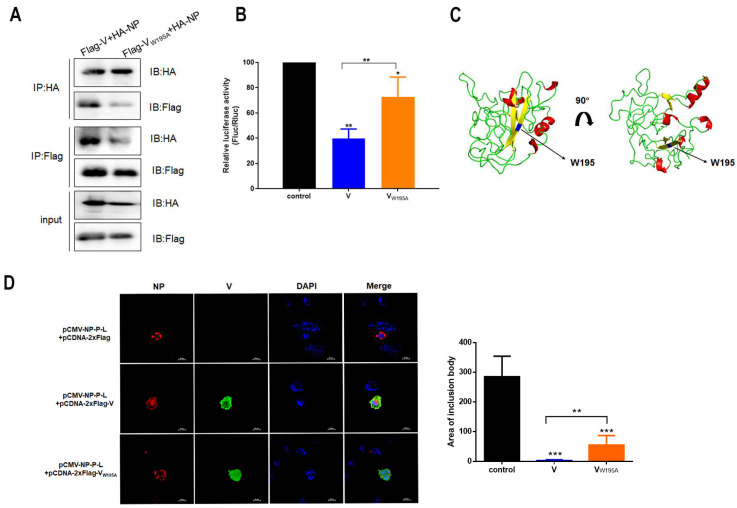
The V mutant regulates MG-encoded reporter activity and alters IB formation in transfected cells. (**A**) Interaction between V_W195A_ and NP. (**B**) Inhibition of luciferase activity by V and V_W195A_ in cotransfected BHK-21 cells. A dual-luciferase assay was performed to measure luciferase activity at 36 h post-transfection. *, *p* < 0.05; **, *p* < 0.01. (**C**) 3D model of V structure built by Swiss-model with crystal structure of the DDB1-Cul4A-Rbx1-SV5V complex (2hye.1.B) as template (helix: red; sheet: yellow; loop: green). W195 residue is shown in blue. (**D**) Effect of V and V_W195A_ on IB formation. DF-1 cells were cotransfected with pCMV-NP-P-L, pCDNA-2xFlag-V or pCDNA-2xFlag-V_W195A_ and analyzed by IFA at 24 hpi. NP (red), V (green) and nuclei (blue) were observed by confocal microscopy; scale bar, 20 μM. The area of IB was measured by ImageJ 1.8.0. **, *p* < 0.01; ***, *p* < 0.001.

To further investigate the role of the W195 residue in V, a mutant plasmid of V (Flag-V_W195A_) in which tryptophan was replaced with alanine was generated. Co-IP experiments were performed in HA-NP and Flag-V_W195A_ cells cotransfected with plasmids. The results revealed a significant reduction in the interaction between the V and NP proteins (Figure 3A). Furthermore, although the activity of the MG reporter was inhibited when it was coexpressed with V and V_W195A_, the inhibitory activity of V_W195A_ was slighter than that of V (Figure 3B). A theoretical 3D-structure prediction of V contained five α-helical structures and four β sheets. The W195 residue was located in one of the β sheets formed by 191–198 aa (Figure 3C).

### 3.3. The V Protein Alters IB Formation in Transfected Cells

NDV infection can cause IB formation in cells that form the core for viral transcription and replication, which is also called the viral factory. The IB mainly includes the viral RNP complex. Since the V protein interacts with NP, whether the V protein affects IB formation was investigated. Immunofluorescence (IFA) assays were conducted by coexpressing NP-P-L with V and V_W195A_. Large IBs were observed when NP-P-L was expressed in DF-1 cells. Coexpression of V led to the redistribution of NP throughout the cytoplasm and inhibited IB formation. In cells coexpressing V_W195A_, IBs were smaller than those in cells not expressing V (Figure 3D). These results suggested that V reduces IB formation and that the inhibitory effect is correlated with W195.

### 3.4. Rescue of the Class II Genotype IX Strain F48E9

**Figure 4:**
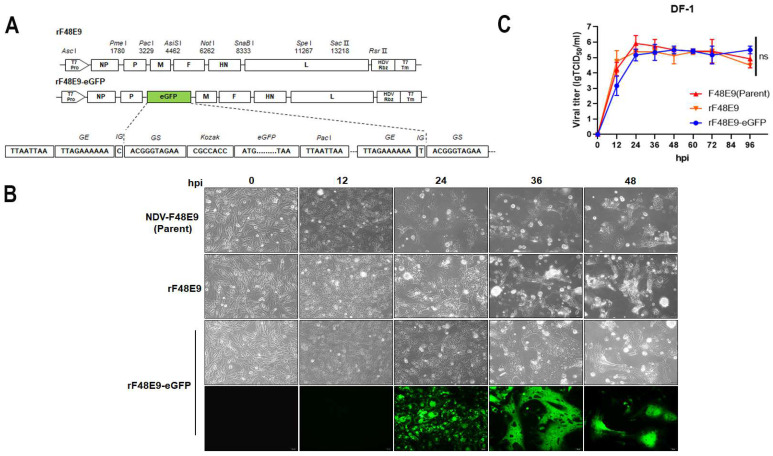
Construction and rescue of rF48E9 and rF48E9-eGFP. (**A**) Construction scheme of full-length plasmids of rF48E9 and rF48E9-eGFP (Pro, promotor; Tm, terminator; GE, gene end; IG, intergene; GS, gene start). (**B**) CPE and eGFP expression in infected DF-1 cells at an MOI of 0.01; scale bar, 50 μM. (**C**) Viral proliferation of infected DF-1 cells at an MOI of 0.01. The data are representative of three independent experiments. ns, not significant.

To understand the effects of V–NP interaction on viral replication and pathogenesis, a full-length infectious clone of the highly virulent strain F48E9 and its clone with an eGFP reporter inserted between the P and M genes were constructed (Figure 4A). eGFP expression was apparently observed at 24 hpi in rF48E9-eGFP. The recombinant viruses caused similar CPEs to those of the parental F48E9 strain in infected DF-1 cells at an MOI of 0.01 (Figure 4B). The growth titer of the two recombinant viruses did not differ from that of the parental F48E9 in the DF-1 cells (Figure 4C). These results indicated that the CPE and replication of the rescued rF48E9 viruses were similar to those of the parental F48E9 virus.

### 3.5. The W195 Mutation of V Increases Viral Replication and IB Formation in Infected Cells

**Figure 5:**
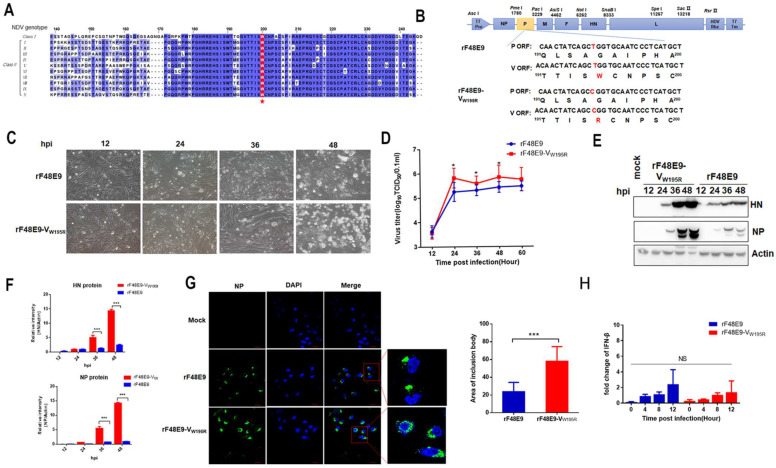
The rescue and growth characteristics of rF48E9-V_W195R_ in cells. (**A**) Comparison of V amino acid sequences in class I and class II genotypes I to X was performed by MegAlign and edited by Jalview. (**B**) Construction scheme of rF48E9-V_W195R_. (**C**) CPE of rF48E9 and rF48E9-V_W195R_ in infected DF-1 cells at an MOI of 0.01. (**D**) Growth kinetics of virus in DF-1 cells infected with rF48E9 and rF48E9-V_W195R_ at an MOI of 0.01. (**E**) The protein expression of NP and HN in DF1 cells was analyzed by Western blot. (**F**) The expression levels of NP and HN in DF1 cells (**E**) relative to that of β-tubulin were analyzed by densitometry. (**G**) IB formation induced by rF48E9 and rF48E9-V_W195R_ infection. DF1 cells were infected with viruses (MOI = 0.01). The area of IB was measured by ImageJ 1.8.0. (**H**) IFN-β mRNA expression in the rF48E9- and rF48E9-V_W195R_-infected DF-1 cells. The relative mRNA expression of β-IFN normalized to that of 28S rRNA was determined by RT-qPCR. Representative data are shown as the means ± SDs (*n* = 3) from three independent experiments. NS, not significant; *, *p* < 0.05; ***, *p* < 0.001.

Since W195 of the V protein is critical for V–NP interaction, whether this residue mutation affects viral replication was studied. W195 was highly conserved in the class I and genotype I to X V proteins of class II NDV (Figure 5A). To generate the W195 mutant virus, the nucleotide “T” at position V583 of the P gene was changed to “C”, resulting in the substitution of W with arginine (R) at position 195 of the V protein. Importantly, this mutation did not alter the amino acid sequence of the P protein (Figure 5B). The CPE caused by rF48E9-V_W195R_ in DF-1 was more severe than that caused by rF48E9 at 36 hpi and 48 hpi (Figure 5C). The growth kinetics of DF-1 cells in rF48E9-V_W195R_ were slightly greater (*p* < 0.05) than those in rF48E9 at 24 to 48 hpi (Figure 5D). Similarly, the expression levels of the HN and NP proteins in rF48E9-V_W195R_ were greater than those in rF48E9 (Figure 5E,F). Furthermore, the size of the inclusion bodies induced by rF48E9-V_W195R_ was significantly greater than that induced by rF48E9 (Figure 5G). There was no significant difference in the level of IFN-β in DF-1 cells induced by rF48E9-V_W195R_ or rF48E9 (Figure 5H). These results suggested that the effect of W195 on viral replication via the V protein was associated with IB formation in the cells.

### 3.6. The W195 Mutation of V Reduces Virulence and Pathogenicity

**Figure 6:**
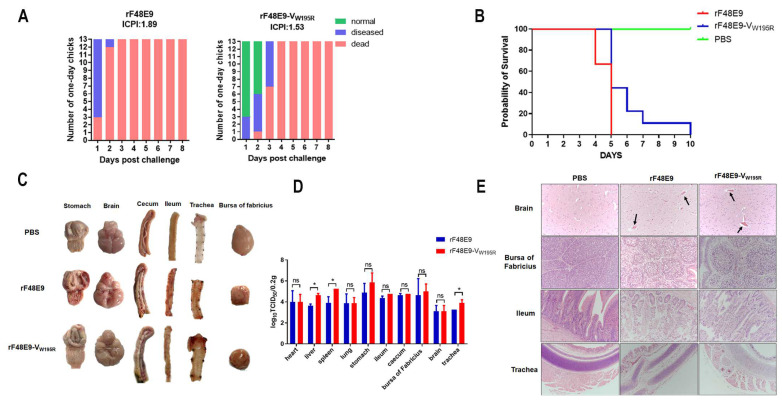
Virulence and pathogenicity of rF48E9-V_W195R_ in vivo. (**A**) ICPI of the recombinant viruses. (**B**) Survival rates of the virus-infected chickens. (**C**) Gross lesions of the organs in the virus-infected chickens. (**D**) Viral replication in chickens. The results are presented as the means ± SDs from three independent experiments. NS, not significant; *, *p* < 0.05; (**E**) HE assay showing the histopathology of the virus-infected chickens. The black arrows indicate vascular cuffing lesions in the brain.

To investigate the role of the V protein W195R mutant virus in vivo, its virulence was measured by ICPI and MDT. The MDT of rF48E9-V_W195R_ was 64 h, which was slightly greater than that of rF48E9 (47 h). The ICPI of rF48E9-V_W195R_ (1.53) was lower than that of rF48E9 (1.89) (Figure 6A). The results indicated that the virulence of the W195R mutant was reduced. Furthermore, three-week-old SPF chickens were challenged with 10^5^ pfu of either rF48E9 or rF48E9-V_W195R_ via the intraocular–nasal route. All the chickens showed diarrhea, depression, cough, asthma and neurological symptoms. The chickens infected with rF48E9 began to die at 3 dpc, and all died at 5 dpc, while the chickens infected with rF48E9-V_W195R_ exhibited delayed mortality with deaths occurring at 5 dpc and all chickens dying at 10 dpc (Figure 6B). Clinical anatomical examinations revealed glandular gastric congestion, hemorrhage and muscular gastric ulcer, cerebral hemorrhage, hemorrhage and necrosis in the cecum and ileum, tracheal congestion and bursa of fabricius atrophy accompanied by mucous and hemorrhage in the rF48E9-challenged chickens. The lesions in the glandular stomach, brain and ileum of the rF48E9-V_W195R_-challenged chickens were less severe (Figure 6C). The tissue viral loads of both viruses replicated well in the chickens with no significant difference in most organs, and the titers of rF48E9-V_W195R_ were slightly greater in the liver, spleen and trachea than those of rF48E9 (Figure 6D). In terms of pathological changes, follicular atrophy and increased interfollicular spaces in the bursa of fabricius, exfoliation of the villous epithelium of the small intestine and desquamated tracheal epithelial cells were slighter in rF48E9-V_W195R_ infection than those in rF48E9 by HE staining. Lymphocyte infiltration and hemorrhage in the brain were observed in both virus infections with no significant difference (Figure 6E). Overall, these findings suggested that the W195R mutation in the V protein decreased the virulence and pathogenicity of NDV in the chickens.

## 4. Discussion

NDV causes highly pathogenic diseases with substantial economic impacts on the poultry industry. Its transcription and replication are mediated by viral RNP complexes, including viral RNA and the viral proteins NP, P and L. The viral non-RNP proteins V of HPIV2 and SeV regulate viral transcription and replication [23,25], but the role of V of NDV is completely unknown. In this study, using an MG system, we determined that the V protein of NDV significantly reduced MG-encoded eGFP expression and Fluc activity compared with the M, W, F and HN proteins. V was found to interact with the NP, inhibiting viral RNA synthesis and IB formation in the cells. Residue W195 of V was found to be crucial for V–NP interaction and IB formation. Using an established infectious clone of the highly virulent F48E9 strain, the rF48E9-V_W195R_ mutant affected viral replication and the pathogenicity of rF48E9 in chickens. These results suggested that the W195 residue of V plays a role in V-NP interaction because of its involvement in multiple aspects in NDV.

The MG system is widely used to study viral transcription and replication, including the roles of viral proteins, the location and boundaries of cis-acting elements, the functional domains of trans-acting proteins, virus-host interactions and the structure and function of viral RNA [29]. Using the MG system, the transcription and replication of paramyxovirus-encoded proteins, especially V, such as SeV, SV5, MeV, HPIV5, NiV and HPIV2, can be regulated [20,21,22,23,24,25]. In our study, two MG systems of NDV with different reporters, eGFP and Fluc, were created to investigate the role of viral non-RNP proteins in viral transcription and replication. Both system assays showed a remarkable inhibitory effect of V on RNA synthesis compared with that of the other viral proteins M, W, F and HN. During MG transfection experiments, the empty vector pCDNA-2xFlag showed a reduction in MG-encoded reporter expression along with an increasing plasmid amount. This might be due to the large amount of DNA transfection stimulating the cell stress response, which limits MG-encoded reporter expression.

Viral V can interact with NP of SeV and HPIV5 [20,23]. Our study showed that NDV V interacts with NP by Co-IP and colocalization experiments. V interacted with NP via its 1-195 aa region. Furthermore, residue W195 of V was identified as a key amino acid for V–NP interaction by protein truncation Co-IP assays. W195 of V is highly conserved among NDV strains and other paramyxoviruses [30]. The ^195^WCNP^198^ of V is conserved among paramyxoviruses, and the W^5^/W^9^/^19^WCNP^22^ motif of the MuV V protein is located upstream of the Cys-rich motif, which is critical for STAT complex formation [31,32]. In addition, the W motif (W-X_3_-W-X_9_-W) of HPIV2 V is critical for the blockade of Toll-like receptor 7 (TLR7)- and TLR9-dependent signaling [30].

IBs are virus-induced compartments for viral transcription and replication in host cells. The NP is essential for IB formation in NDV [12]. Our study revealed that IB formation could be disrupted by V expression in NP-P-L-cotransfected DF-1 cells, while the inhibitory effect of the mutant V_W195A_ was diminished. The inhibition of IB formation was mediated by V–NP interaction, leading to the distribution of NP throughout the cytoplasm and, subsequently, impairing IB formation. A similar study revealed that the HPIV3 M protein reduces IB formation by interacting with its N protein [33].

Since the reverse-genetics system of NDV was established in 1999 [34], it has penetrated various research areas, including the identification of virulence factors and the clarification of gene functions as well as the insertion of exogenous genes as vaccine vectors to form multivalent vaccines [4]. At present, more than thirteen strains of different genotypes in the class II and class I categories have been rescued [35]. The F48E9 strain is a highly virulent strain of class II genotype IX isolated in northern China in 1948 [36]. This strain is subsequently used as the standard challenge strain for vaccine evaluation [37]. The reverse-genetics system of F48E9 was first established in genotype IX strains. This approach will not only provide a powerful platform but also promote the understanding of the mechanism of this virus’s pathogenesis.

The viral replication and virulence of rescued rF48E9 were the same as those of parental F48E9. However, the mutant rF48E9-V_W195R_ increased viral replication and IB formation in DF-1 cells, suggesting that V is a negative regulator of viral replication and IB formation. The V protein can antagonize host innate immunity, especially IFN, thereby favoring viral replication [38,39,40]. To determine whether the inhibitory effects of V on viral replication are mediated by host IFN expression, we assessed the levels of IFN-β induced by rF48E9-V_W195R_ and rF48E9 in DF-1 cells using RT-qPCR. There was no significant difference in the levels of IFN-β at early time points, and both viruses caused low IFN-β mRNA levels in DF-1. This result is consistent with a previous report [41]. Therefore, the inhibitory effect of V might not be related to host IFN during early infection because of V–NP interaction.

The virulence of rF48E9-V_W195R_ decreased in response to MDT and ICPI. In infected chickens, the mutant rF48E9 retarded the onset of disease and the time of death and reduced pathological changes. Moreover, the viral loads of rF48E9-V_W195R_ were greater in trachea, liver and spleen tissues than those of rF48E9. The viral replication of rF48E9-V_W195R_ increased in the cells and chicks, but its pathogenicity decreased. It is possible that the pathogenicity of rF48E9-V_W195R_ may be reduced by host factors, which may interact with V_W195R_ to impair the regulatory balance of V during host-virus interactions in vivo. This unsolved puzzle needs further investigation.

In summary, the V of NDV, a viral self-regulator, was identified and verified by established MG and reverse-genetics systems. Residue W195 of V plays crucial roles in viral RNA synthesis, V–NP interaction, IB formation, replication and pathogenicity both in vitro and in vivo. This study provides insight into the self-regulation of V in paramyxoviruses.

## Data Availability

All data generated and analyzed in this research are included in the article.

## Supplementary Materials

The following supporting information can be downloaded at: https://www.mdpi.com/article/10.3390/v16040584/s1, Table S1: Primers used in this study.
