# Supplementary material for: The W195 Residue of the Newcastle Disease Virus V Protein Is Critical for Multiple Aspects of Viral Self-Regulation through Interactions between V and Nucleoproteins
Source: Viruses. 2024 Apr 10;16(4):584. doi: 10.3390/v16040584 (PMC11054343; doi:10.3390/v16040584)
Supplement: Supplementary file 1 [file viruses-16-00584-s001.zip › viruses-2932163-supplementary.pdf]

Table S1. Primers used in this study

Primer name	Primer sequence 5'---3'
eGFP-F	CC***ATCGAT***ATGGTGAGCAAGGGCGAGGAG
eGFP-R	ACAT***GCATGC***TTACTTGTACAGCTCGTCCATGCC
Fluc-F	CC***ATCGAT***ATGGAAGATGCCAAAAACATTAAG
Fluc-R	AT***GCATGC***ACTTTACACGGCGATCTTGCCGCCCTTC
LaSota-NP-F	CG***GAATTC***ATGTCTTCCGTATTTGATGAGTACGA
LaSota-NP-R	CCG***CTCGAG***TCAATACCCCCAGTCGGTGTCGTTA
LaSota-P-F	CG***GAATTC***ATGGCCACCTTTACAGATGCAGAGA
LaSota-P-R	CCG***CTCGAG***TTAGCCATTTAGAGCAAGGCGCTTG
LaSota-L-F	CATG***CCATGG***CGAGCTCCGGTCCTGAAAG
LaSota-L-R	GG***ACTAGT***TTAAGAGTCACAGTTACTGTAATATCC
T7-F	CG***GAATTC***ATGAACACGATTAACATCGCTAAG
T7-R	CCG***CTCGAG***TTACGCGAACGCGAAGTCCGACTC
LaSota-V-F	CG***GGATCC***ATGGCCACCTTTACAGATGCAG
LaSota-V-R	CC***CTCGAG***TTACTTACTCTCTGTGATATC
LaSota-M-F	CG***GAATTC***ATGGACTCATCTAGGACAATTGGGC
LaSota-M-R	GC***TCTAGA***TTATTTCTTAAAAGGATTGTATTTGGC
LaSota-W-F1	CG***GGATCC***ATGGCCACCTTTACAGATGCAG
LaSota-W-R1	CC***CTCGAG***TCACGTCTGTGCCCTGGTTTC
LaSota-W-F2	AATGCTAAAAAGGGGGCCCATGGTCGAGCCCCCAA
LaSota-W-R2	GGGCCCCCTTTTTAGCATTGGAC
LaSota-F-F	***GGATCC***ATGGGCTCCAGACCTTCTACCAAG
LaSota-F-R	cc***CTCGAG***TCACATTTTTGTAGTGGCTCTC
LaSota-HN-F	***GGATCC***ATGGACCGCGCCGTTAGCCAAG
LaSota-HN-R	GC***TCTAGA***CTAGCCAGACCTGGCTTCTCTAAC
LaSota-NP-F	***GAATTC***ATGTCTTCCGTATTTGATGAGTACG
LaSota-NP-R	***CTCGAG***TCAATACCCCCAGTCGGTGTCG
LaSota-V_{W195R}-F1	CG***GGATCC***ATGGCCACCTTTACAGATGCAG
LaSota-V_{W195R}-F2	CAACTATCAGCGCCTGCAACCCCTCATG
LaSota-V_{W195R}-R1	CC***CTCGAG***TTACTTACTCTCTGTGATATC
LaSota-V_{W195R}-R2	CATGAGGGGTTGCAGGCGCTGATAGTTG
LaSota-$V_{1\text{-}221}$-F	CG***GGATCC***ATGGCCACCTTTACAGATGCAG
LaSota-$V_{1\text{-}221}$-R	***CCGCTCGAG***TTAACAGGTGGCTGGACATGATCCG
LaSota-$V_{1\text{-}198}$-F	CG***GGATCC***ATGGCCACCTTTACAGATGCAG
LaSota-$V_{1\text{-}198}$-R	***CCGCTCGAG***TTAGGGGTTGCACCAGCTGATAG
LaSota-$V_{1\text{-}197}$-F	CG***GGATCC***ATGGCCACCTTTACAGATGCAG
LaSota-$V_{1\text{-}197}$-R	CCG***CTCGAG***TTAGTTGCACCAGCTGATAGTTG
LaSota-$V_{1\text{-}196}$-F	CG***GGATCC***ATGGCCACCTTTACAGATGCAG
LaSota-$V_{1\text{-}196}$-R	CCG***CTCGAG***TTAGCACCAGCTGATAGTTGTG
LaSota-$V_{1\text{-}195}$-F	CG***GGATCC***ATGGCCACCTTTACAGATGCAG
LaSota-$V_{1\text{-}195}$-R	CCG***CTCGAG***TTACCAGCTGATAGTTGTGAC
LaSota-$V_{1\text{-}194}$-F	CG***GGATCC***ATGGCCACCTTTACAGATGCAG
LaSota-$V_{1\text{-}194}$-R	CCG***CTCGAG***TTAGCTGATAGTTGTGACTCC

LaSota-V_{1-193}-F	CG***GGATCC***ATGGCCACCTTTACAGATGCAG
LaSota-V_{1-193}-R	CCG***CTCGAG***TTAGATAGTTGTGACTCCTCCC
LaSota-V_{1-192}-F	CG***GGATCC***ATGGCCACCTTTACAGATGCAG
LaSota-V_{1-192}-R	CCG***CTCGAG***TTAAGTTGTGACTCCTCCCATTG
LaSota-V_{1-191}-F	CG***GGATCC***ATGGCCACCTTTACAGATGCAG
LaSota-V_{1-191}-R	CCG***CTCGAG***TTATGTGACTCCTCCCATTGTCC
LaSota-V_{1-183}-F	CG***GGATCC***ATGGCCACCTTTACAGATGCAG
LaSota-V_{1-183}-R	CCG***CTCGAG***TTATATGCTGTGTTCACGTCTGTG
LaSota-V_{1-175}-F	CG***GGATCC***ATGGCCACCTTTACAGATGCAG
LaSota-V_{1-175}-R	*GCC**CTCGAG***TTATGGTTTCCAGGGGCGGCCTTG
rF48-P_{W195R}-F1	AGCTTT***GTTTAAAC***AGAACCAAAGACATTAGAAAAAA ATACG
rF48-P_{W195R}-R1	TGCC***TTAATTAA***GGTTGCGCGATTATTTAATGAGATAG AGGAGG
rF48-P_{W195R}-F2	ACAACTATCAGCCGGTGCAATCCCTCATG
rF48-P_{W195R}-R2	CATGAGGGATTGCACCGGCTGATAGTTGT
chicken 28S qPCR-F	GGTATGGGCCCGACGCT
chicken 28S qPCR-R	CCGATGCCGACGCTCAT
chicken IFN-β qPCR-F	GCTCACCTCAGCATCAACAA
chicken IFN-β qPCR-R	GGGTGTTGAGACGTTTGGAT
Fluc (vRNA)- reverse transcription	ACG ATA AAA GGC GAA GGA GCA
Fluc (cRNA)- reverse transcription	ATG ACG AGA CTA CAC TCA AG
Fluc-qPCR-F	5’-CGCACATATCGAGGTGGACA-3’
Fluc-qPCR-R	5’-GCAAGCTATTCTCGCTGCAC-3’
oligonucleotide linker	ACTTGGCGCGCCAAGCTTTGTTTAAACGGCCCTTAATT AAGGACGCGATCGCGTATAAGAATGCGGCCGCTAAAC TATGCTACGTACCGGACTAGTCCTCCCCGCGGGGATTC GGTCCGAAT
F48E9-Asc-T7-F	AGTT***GGCGCGCC***TAATACGACTCACTATAGGGACCAA ACAGAGATTCTGTGAGGTACGATAAAAAGC
F48E9-Pme1780-F	AGCTTT***GTTTAAAC***AGAACCAAAGACATTAGAAAAAA ATACG
F48E9-Pme1780-R	CCGGCGCGCC***GTTTAAAC***TGTTGATGGGTCAAGTTGTG CCTGTGTTG
F48E9-Pac3229-F	TGCC***TTAATTAA***TCCTGCAACATTAATGATTAAGAAAA AATACGG
F48E9-Pac3229-R	TGCC***TTAATTAA***GGTTGCGCGATTATTTAATGAGATAG AGGAGG
F48E9-AsiS4462-F	ACGATT***GCGATCGC***TTATAGTTAGCTCACCTGTCTATC
F48E9-AsiS4462-R	ACGATT***GCGATCGC***GACAGATCATTATTTGGCGCCGT GGTG
F48E9-Not6262-F	ATAAGAAT***GCGGCCGC***CTGTCAATTAGAAGAATTAAG

	AAAAAACTACC
F48E9-Not6262-R	ATAGTTTA***GCGGCCGC***AATTCACACACAAATTGCTCTTGGAGATAC
F48E9-delRsr6493-F	TTCGAACAGCAGTCTTACTTTTAATAGTAGTGACCTTTTC
F48E9-delRsr6493-R	GTCACTACTATTAAAAGTAAGACTGCTGTTCGAAATACCAAGCGCCATGTATTTTTCG
F48E9-SnaB8333-F	ATCAGG***TACGTA***GTAGTGAGATACGAGACAAAGCAACTCAC
F48E9-SnaB8333-R	ATCAGG***TACGTA***TATTTTTTCTTAATCAAGTGACTATTGGCAAG
F48E9-Spe11267-F	GG***ACTAGT***CCACCTGGTAATGGAGATTGGGCCAG
F48E9-Spe11267-R	GG***ACTAGT***TAAGATGTTAGTCATAATGCTAGGAC
F48E9-Sac13218-F	TCC***CCGCGG***AAGAAAAATGTTCAGTACTCACTGAG
F48E9-Sac13218-R	TCC***CCGCGG***ATAAGCCTCTTATTTTCGGGATTTC
F48E9-delRsr13828-F	GGGCCAACCCCGACACAGTTCCTGAATTCAGTC
F48E9-delRsr13828-R	GACTGAATTCAGGAACTGTGTCGGGGTTGGCCCGAAATGTCGCTGTGGGGGGTTCATC
F48E9-Rsr-Rbz-R	GGT***CCGGAC***CGCGAGGAGGTGGAGATGCCATGCCGACCCACCAAACAAAGATTTGGTGAATGACAGAAC
Pac-GFP-F	CC***TTAATTAA***TTAGAAAAAACACGGGTAGAACGCCACCATGGTGAGCAAGGGCGAGGAGCTGTTC
CE-PacI-GFP-R	TCATTAATGTTGCAGGA***TTAATTAA***TTACTTGTACAGCTCGTC

The restriction enzyme sites are in italic font and bold.
